# Breaking In through Critical Windows: *p,p*′-DDE May Alter Fetal Neurodevelopment

**Published:** 2007-03

**Authors:** Julia R. Barrett

DDT has been widely used to control mosquito-borne malaria since the late 1940s. The compound and metabolites such as *p,p*′-DDE linger in the environment for decades; even in areas where DDT has been banned, these neurotoxic chemicals are still detected in human blood, fat, breast milk, and umbilical cord blood. Researchers examined the possibility that prenatal exposure to *p,p*′-DDE damages early neurodevelopment, and present the first evidence that exposure during a critical window of development adversely affects infant psychomotor development.**[*EHP* 115:435–439; Torres-Sánchez et al.]**.

From January 2001 to June 2005, 1,585 reproductive-age women in the State of Morelos, Mexico, where DDT had been used for malaria control until 1998, were invited to join the prospective cohort study. Each woman choosing to participate provided a blood sample and information about sociodemographic characteristics, obstetric and gynecologic history, alcohol and tobacco use, occupation, and previous pesticide use.

Once a woman became pregnant, the researchers conducted in-home visits each trimester to collect a blood sample and data on her pregnancy, weight, and diet. After the woman gave birth, they evaluated her child at 1, 3, 6, and 12 months of age, focusing on health, feeding, growth, and cognitive and psychomotor development. The researchers also tested maternal intelligence and assessed the home environment by observing factors such as parent–child interaction and available toys. Data were available for 244 mother–child pairs.

*p,p*′-DDE was detected in all maternal blood samples. Concentrations were the highest in the third trimester, but analyses revealed that only first-trimester concentrations were associated with impaired psychomotor development. This association remained after controlling for maternal intelligence and the home environment; breastfeeding appeared to have a slight protective effect.

A subset of 105 maternal blood samples were also tested for lead. Because maternal lead concentrations were not available for all infants, lead exposure could not be completely excluded as contributing to effects correlated with first-trimester *p,p*′-DDE exposure. However, the low negative correlation between the two neurotoxicants made it unlikely that the effects observed were either amplified or masked by lead. There did not appear to be an association between prenatal *p,p*′-DDE exposure and cognitive development.

These findings add to the growing evidence that DDT metabolites in a mother affect her child’s psychomotor development during infancy. The researchers suggest that prenatal *p,p*′-DDE exposure needs further attention, even in countries where DDT has not been used for decades.

## Figures and Tables

**Figure:**
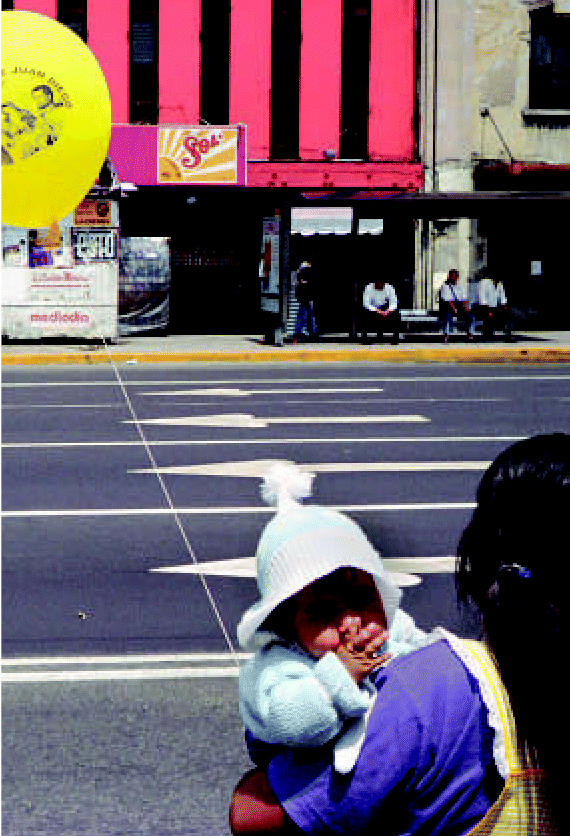
Back tracks Long-ago pesticide spraying can still affect today’s children.

